# Research‐practice partnerships in education: Benefits, challenges, methodological considerations and key enablers for change

**DOI:** 10.1111/bjep.12785

**Published:** 2025-05-29

**Authors:** Sarah McGeown, Simon Sjölund

**Affiliations:** ^1^ Moray House School of Education and Sport University of Edinburgh Edinburgh UK; ^2^ School of Education, Culture and Communication Mälardalen University Västerås Sweden

**Keywords:** collaboration, co‐production, education, research, research‐practice partnership, systems

## Abstract

**Background:**

Research‐practice partnerships reflect collaborations between researchers and practice‐based professionals to co‐produce new research knowledge and are often cited as one way to narrow the widely recognized gap between research and practice. Within the context of education, research‐practice partnerships draw upon the knowledge, expertise and experience of researchers and practice professionals with the aim of co‐producing methodologically robust and educationally relevant research aligned with priorities of practice. Over the last decade, this methodological approach has been gaining traction, yet it is not without its challenges and methodological considerations, which need to be understood and carefully navigated as this trajectory seems set to continue.

**Aims:**

This article reviewed the benefits, challenges and methodological considerations associated with research‐practice partnerships, and proposes four key enablers—infrastructure, funding, training and incentives—for research‐practice partnerships to be optimally introduced, embedded and sustained.

**Methods:**

Drawing upon the research literature and diverse examples of infrastructure, funding, training and incentives across different European contexts, specifically Sweden, Norway, Germany, England and Scotland, this article aimed to synthesize academic and real‐world insights to advance knowledge and thinking in this area.

**Conclusions:**

This article provides a contemporary account of key enablers that can initiate, develop, and sustain research‐practice partnerships, drawing on insights from the research literature and illustrative examples of existing structures. In doing so, it aims to make a significant contribution to knowledge, thinking and potentially practice, as we work more collectively and productively together to reduce education inequalities and improve the educational experiences and outcomes of children and young people.

## INTRODUCTION

National reforms across the Western world are increasingly emphasizing the use of academic research to inform educational practice (e.g., Farley‐Ripple et al., 2018), recognizing the limited impact that research has had on educational policy and practice to date and the gap which continues to exist between research and practice (Penuel et al., 2020). Collaborative approaches, specifically co‐production between university‐based researchers and external stakeholders/practitioners, are seen as promising for bridging this divide (Penuel et al., 2020). Such collaboration is argued to produce more timely and relevant research, and practical pedagogies and innovations to enhance student learning (Coburn & Penuel, 2016).

Co‐production, defined in this article as the collaborative generation of new research knowledge involving academic researchers and non‐academic stakeholders, has emerged as a key strategy for addressing educational challenges. Co‐production has been conceptualized and enacted in various ways, yet a promising way of working with co‐production is research‐practice partnerships. In a seminal article, Farrell et al. (2021) describe research‐practice partnerships as: ‘A long‐term collaboration aimed at educational improvement or equitable transformation through engagement with research’ (p. 4). Research‐practice partnerships (RPPs) are defined by their intentional approach to organizing work, combining diverse expertise and employing strategies to shift power relations to ensure that all participants have a meaningful role in decision‐making and knowledge production. Further, RPPs are characterized by research as the core activity, with the research agenda typically driven by priorities identified from those working in practice. By working in this way, research‐practice partnerships focus on key educational challenges, using rigorous research to address these.

Research‐practice partnerships use intentional strategies to foster collaboration (Farrell et al., 2021), often relying on rules, roles, routines and protocols to structure their interactions. There is no single ‘best’ model for research‐practice partnerships; the most effective partnerships enable individuals across diverse sectors, but with shared interests and priorities, to work effectively towards more educationally relevant research, as well as educational improvement (Farrell et al., 2021). Research‐practice partnerships not only foster mutual learning, but also ensures the relevance, credibility and legitimacy of research by engaging these diverse knowledge systems (Bandola‐Gill et al., 2023). Research‐practice partnerships vary in goals, composition, research approaches and funding sources (Farrell et al., 2021). Goals can be narrowly focused, such as improving literacy attainment, or broadly aimed at addressing systemic inequities. Their composition ranges from small collaborations to multi‐tiered networks involving schools, universities, national and/or community organizations and/or others. Research approaches differ, including co‐design, evaluation, and improvement science, while funding shapes their scope and sustainability. This diversity allows research‐practice partnerships to address unique challenges, however effective partnerships require strategic organization to manage competing demands.

A recent systemic review of research‐practice partnerships (Sjölund et al., 2022) provided important insights into different types of research utilized within partnerships, different forms of partnership and the different ways in which research is used. Within the present article, the authors intend to expand knowledge and thinking by drawing upon research and grey literature to provide a contemporary account of research‐practice partnerships, from theoretical, empirical and practical positions. First, the authors synthesize previous research literature to highlight key benefits, challenges and methodological considerations associated with research‐practice partnerships, before advancing and expanding previous work (McGeown, 2023) on key enablers for change. In doing so, the authors draw upon both research and grey literature to highlight key enablers to support the development and sustainability of research‐practice partnerships, namely infrastructure, funding, training and incentives. By providing examples across a number of European contexts, the authors provide diverse insights into the ways in which research‐practice partnerships can, and are, reshaping our research ecosystem and closing the gap between research and practice within education.

## BENEFITS

Multiple benefits have been cited in relation to research‐practice partnerships, most notably is their potential to narrow the widely recognized gap between research and practice (Penuel et al., 2020). For example, research‐practice partnerships are more likely to lead to research which is more closely aligned with practice partner's interests, needs and priorities, thus increasing the likelihood of research use and impact (Coburn & Penuel, 2016). Research‐practice partnerships can also increase the likelihood of acceptability and feasibility of research‐informed interventions during their creation and implementation in practice (Cai & Hwang, 2021; Scanlon et al., 1994; Snow, 2015).

Further, the co‐production of research also increases opportunities for a much wider range of individuals to contribute to the pursuit of new knowledge to improve educational thinking, knowledge and practice. In doing so, research‐practice partnerships democratize University‐based research (Sjölund, 2023), providing practitioners with the opportunity to contribute to the development of knowledge, rather than simply being consumers of it (Bevins & Price, 2014). Through positive and productive collaborative experiences, research‐practice partnerships can also improve professionals' attitudes towards university‐based research (Ross & Bruce, 2012), in addition to supporting their professional learning and development (Steel et al., 2021) and the professional learning and development of researchers (McGeown et al., 2023).

Finally, through building positive, productive, and trusting relationships, researchers and practitioners can support the individual aims of each other. For example, researchers can support practitioners by providing useful research insights to support schools, districts or education organizations facing budget cuts and in need of independently produced data analyses or new research to inform decision‐making (López Turley & Stevens, 2015). On the other hand, schools, districts and education organizations can provide University‐based researchers with access to their data, supporting researchers' efforts to achieve positive educational impact (e.g., Farley, 2019; Moeller et al., 2018).

## METHODOLOGICAL CONSIDERATIONS AND CHALLENGES

Entering into research‐practice partnerships to co‐produce educational research without sufficient knowledge of the methodological considerations can have negative consequences both for the research project and those involved. Indeed, Baum (2000) warns of researchers becoming overzealous and uncritical about research‐practice partnerships and exaggerating their potential, without sufficient understanding of the methodological considerations or challenges involved. For example, researchers and practice professionals may have different perspectives of what research is, or what research is needed, and without reaching a consensus prior to commencing projects, outcomes may satisfy neither (London et al., 2018). Further, a clear and agreed understanding of roles (Farrell et al., 2019) and ways of working (McGeown et al., 2023) is essential for positive collaboration and outcomes. Yet, even well‐suited designs can face challenges, as improvement efforts in complex educational systems require ongoing adaptation to dynamic contextual factors. Further, some designs may fail if participants lack commitment or confidence in their roles (Coburn & Penuel, 2016; Sjölund & Lindvall, 2023).

Further, the co‐production of research through research‐practice partnerships requires a methodological shift from traditional forms of university‐based research, which have historically been researcher‐led, to more collaborative research practices, which draw upon the knowledge, experience and expertise from both researchers and practice professionals (McGeown et al., 2023). This shift can lead to new interests and agendas for university‐based research, which can require new ways of thinking, researching and communicating, and therefore an openness to different perspectives and new ways of researching is vital (Coburn & Penuel, 2016).

Indeed, research practice partnerships can have an impact on researchers' research agendas, processes and outputs (Arce‐Trigatti et al., 2018). It can require increased time, compromises and a willingness to learn and adopt new ways of researching to create quality relationships, characterized by trust and respect, and which navigate potential power imbalances (Cooper et al., 2020; Skipper & Pepler, 2021; Snow, 2015). Research‐practice partnerships to co‐produce research also require those experienced of working in very different contexts, with different responsibilities, priorities and time pressures (Steel et al., 2021) to work together, and therefore an appreciation of others' other responsibilities and contexts can also be beneficial (Beveridge et al., 2018). While research‐practice partnerships hold great promise, they also face challenges associated with staff turnover, mismatched timelines and the need to engage decision‐makers with the authority to act on findings (Farrell et al., 2019).

Further, developing a shared language and vocabulary to communicate effectively across research and practice boundaries is also essential for effective collaborative work (Beveridge et al., 2018; London et al., 2018; Steel et al., 2021). The terminology associated with psychological and educational research and practice often differs, yet continues to evolve (e.g., Bottini et al., 2024) in ways which are more likely to foster positive communication between those in research and practice. Beyond the use of specific terminology, language reflects individuals' stances, from a focus on more theoretical, methodological or abstract thinking from researchers to more policy, practical and contextual considerations of professionals (Bevins & Price, 2014)—communicating across boundaries is an important element for establishing and sustaining effective relationships.

Finally, diverse perspectives of the value and use of research and evidence to inform education vary considerably (Biesta, 2007; Goldacre, 2013) and these different perspectives need to be understood and navigated. Effective outcomes from research‐practice partnerships require those leading them to understand the distinct knowledge bases which researchers and professionals have, for example, theoretical and empirical knowledge and methodological expertise (researchers) or knowledge of policy, curriculum, practice, contextual and/or local factors (professionals), both of which can influence outcomes (Cai & Hwang, 2021; Joyce & Cartwright, 2020; Moir, 2018).

## ENABLERS TO INITIATE, DEVELOP AND SUSTAIN RESEARCH‐PRACTICE PARTNERSHIPS

Over the last two decades, there has been rapid growth in research‐practice partnerships internationally. Those most commonly cited have originated within the United States (Sjölund et al., 2022), for example, the Strategic Education Research Partnership, funded by the Institute of Education Sciences (Arce‐Trigatti et al., 2018). However, there is a growing trajectory of their introduction and application in other international contexts and this review reflects on key enablers for change, offering contemporary examples from different European contexts to exemplify different ways in which these enablers may be introduced and embedded in practice.

For research‐practice partnerships to achieve their potential in transforming and improving the quality, relevance and impact of educational research, either at national or local levels, four key enablers for systemic change are proposed: infrastructure, funding, training and incentives. Given the diversity of sources which can, and do, inform research‐practice partnerships, these four enablers were identified by drawing upon research literature and grey literature, in addition to practical knowledge and experience. While there is overlap, for example, funding required to support infrastructure or provide training, each enabler is considered sufficiently important and distinct to discuss independently.

### Infrastructure

Co‐produced research is often dependent upon structures (i.e., clear communication channels and shared online or in‐person spaces to afford collaborative work, in addition to transparent opportunities for others' contribution and involvement), to initiate and sustain research‐practice partnerships throughout the entire research cycle, from conceptualization to completion and dissemination. Given the diversity of ‘research users’ (e.g., policy‐makers, school leaders, teachers, education professionals, families, national and community education organizations), infrastructure needs to be flexible to support and sustain a diversity of relationships across different settings and projects, while sufficient parameters are needed support individuals to collaboratively and effectively reach a shared goal (Farrell et al., 2019). Further, both researchers and practice professionals need to be aware of the different types of infrastructure available to them, and see the value and relevance of it for their own professional needs, in order to meaningful engage with it. Infrastructure which is poorly known, or which does not sufficiently serve the interests or needs of its intended users is likely to remain dormant, which may, correctly or incorrectly, be taken as an indication of a lack of interest for meaningful engagement.

Policy directives and funding schemes promoting collaboration between research and practice have led to new forms of infrastructure being developed to initiate and sustain research‐practice partnerships at national (e.g., state, albeit adapting to local conditions) and local (e.g., regional/project based) levels. The benefits of national infrastructure are opportunities to provide high‐quality training and sustained involvement and engagement, for example, beyond project funding. In recent years, an increase in the use and familiarity of technology to support work, for example, shared online documents, folders and meetings, has significantly facilitated research‐practice partnership infrastructure, affording opportunities to bring together individuals who are geographically dispersed and working within different organizations and sectors to work productively in time and cost‐efficient ways (McGeown et al., 2023).

To illustrate different forms of infrastructure, examples from Norway, Sweden, Germany, England and Scotland are provided to exemplify infrastructures at national and local levels First, in Norway, policies such as the ‘Desire to Learn’ White Paper, have bolstered partnership initiatives by embedding collaborative research into systemic frameworks for professional learning and local development. For instance, the national Scheme for Local Competence Development facilitates long‐term collaborations between universities, schools and kindergartens and emphasizes equitable partnerships rooted in local needs, supporting co‐development of solutions to challenges in practice. The MOVE‐P project is one example which emerged under this scheme and focused on enhancing student motivation and participation combining deductive (research‐based) and inductive (practice‐based) strategies, enabling both researchers and practitioners to contribute their knowledge, experience and expertise.

In Sweden, the 1997 revisions of the Higher Education Act marked a shift from ‘informing about activities’ to actively promoting ‘collaboration with the surrounding society’, leading to the establishment of regional development centres (regionalt utvecklingscentrum, RUC). These centres have been designed to strengthen ties between educational development, research and teacher education, and at Mälardalen University (MDU) in Sweden, the RUC facilitates collaboration by bringing researchers and local practitioners together to co‐develop and refine education projects, which are then jointly led by researchers and practitioners as co‐principal investigators (Co‐PIs). This example illustrates how national policies can lead to infrastructure shifts, for example, the introduction of regional centres to bridge the research‐practice gap through structured and meaningful partnerships.

Meanwhile in Scotland, the Scottish Government's School Research Plan (2023–2026) has set out national priorities to support the government to deliver research informed policy making within schools. Related to research‐practice partnerships, objectives include cross‐sector collaboration across university, public bodies and professional networks, and improving knowledge exchange between policy analysts and users of research across government, academia and educational practice (Scottish Government, 2023). More recently, from 2025, Scotland's Centre for Teaching Excellence will be launched, hosted by a Scottish university (University of Glasgow) to support Scotland's teachers to develop their practice, drawing upon research, among other knowledge sources, to achieve this (Scottish Government, 2024).

While in other contexts, knowledge brokers, that is intermediaries trained to strengthen the relationship between research and practice, have become key to infrastructure. For example, Germany's emphasis on strengthening interdisciplinary collaboration and building capacity for evidence‐informed education led to Peers4Practice, launched in 2023, which aims to strengthen the exchange of educational research and school practice by creating tandem teams (an education researcher and teacher) to work closely together over a sustained period. In doing so, research‐practice infrastructure is aimed to be supported by cohorts of individuals who can act as brokers between educational research and school‐based practice.

Finally, in England, the Education Endowment Foundation's (EEF) Research Schools Network, launched in 2016, represents a sustained national collaboration of schools (*n* = 33) committed to using research evidence to improve educational practice. This network provides support for schools to access, understand and apply research evidence to support the quality of their teaching and learning, and extend this knowledge to other schools (Research Schools Network, 2024). For example, since 2016, over 11,000 schools are reported to have accessed support from their local research school, highlighting a different model of infrastructure to bridge the gap between educational research and practice.

It is essential that both researchers and practice professionals are more aware of the diverse types of infrastructure that exist within their own context, but also have the opportunity to make a meaningful contribution to it, critically reflecting on its current value and use, and being actively involved in shaping it for more effective and productive research‐practice partnerships. The types of infrastructure detailed here are intentionally diverse, to illustrate different ways in which connections between research and practice have been fostered and are being sustained. The original motives, aims, and scope of each form of infrastructure vary—with research‐practice partnerships (as defined within this article) not necessarily being their central or original aim. The development of new, or expansion of existing, infrastructure, which intentionally seeks to foster and sustain research‐practice partnerships as a central aim, is essential if future work in this area is to progress optimally.

### Funding

Co‐produced research typically requires the input and expertise of multiple individuals working across distinctly different organizations; therefore, securing funding for involvement is essential. In relation to research project funding, funding applications are typically led by university‐based researchers, and funding is typically awarded to the University, which can have implications for power dynamics, decision‐making processes and accountability (Cooper et al., 2020; Penuel et al., 2015). Funders that directly allocate funding to the organization involved, in addition to universities, may be one route to more equitable collaboration. However, these could create additional administrative burdens and have consequences for reporting mechanisms, so may not be feasible in practice. Further, funding opportunities are available for projects led by non‐academic organizations for collaborative work, albeit typically without research as the primary focus. For example, in the United Kingdom, project funding applications to Arts Council England or Creative Scotland are led by non‐academic organizations but can have academic input. These different types of funding applications and forms of funding may help to counter embedded power imbalances between research and practice, where applications are led by the sector with the greatest experience and expertise in the area. Yet beyond these distinctly different types of funding for discrete projects or activities, co‐funding or national/state level funding, particularly over a long period, is arguably essential to develop and sustain research‐practice partnerships required for long‐term change (McGeown, 2023; Schlicht‐Schmälzle et al., 2024).

For example, in Germany and Sweden, funding initiatives to integrate research and practice through partnerships (Schlicht‐Schmälzle et al., 2024) have had considerable support from their Ministries of Education (e.g., BMBF; Germany; Ministry of Education and Research, Sweden). In both countries, collaborative structures have been designed not only to conduct research but also to ensure the transfer of research knowledge into educational practice, thereby creating sustainable systems for improving educational outcomes. For example, in Sweden, in 2017, the Swedish government launched the Development, Learning, and Research (ULF) agreement (Government decision, U2017/01129/UH), tasking four universities with coordinating a national initiative to narrow the gap between research and practice through a co‐funding model. Involving all Swedish universities with teacher education programs, the ULF aims to establish sustainable collaboration models between universities and schools and strengthen the scientific foundation of educational practice, as required by Swedish educational law. While in Germany, funded meta‐projects, for example Peers4Practice, play a central role in ensuring that the knowledge generated through these collaborations is effectively applied in real‐world school contexts.

While in the United Kingdom, the Research Excellence Framework (REF) a UK‐wide assessment of research quality, which informs the allocation of approximately £2 billion per year of public funding for UK universities' research, is incentivizing research‐practice partnerships by increasingly funding to universities on the basis of ‘impact’ beyond academia (from 20% in 2014 to 25% in 2021) (Kerridge, 2023). Further, in the next assessment exercise (REF, 2029), funding will be allocated on the basis of ‘engagement and impact’ rather than solely ‘impact’—an important distinction. While the definition of ‘engagement’ is still to be provided, it may reflect, in part, engagement with non‐academic partners to conduct research, which differs from ‘impact’ which can be achieved without non‐academic partners.

At a local/project‐based level, the UK's Economic and Social Research Council (ESRC) is also driving forward this approach by increasingly prioritizing the allocation of project funding on the basis of partnerships with policy and/or practice. In doing so, it encourages University‐based researchers to work with research users/stakeholders to conceptualize, conduct and communicate joint projects. For example, the ESRC Education Research Programme, 2021–2026, funded several university‐led projects based on partnerships between researchers, educators (and in some instances policy makers) to conduct educationally relevant and impactful research (Economic and Social Research Council, 2025). Current ESRC funding is now being allocated to develop and expand the UK policy to research infrastructure (UKRI, 2025) to close the gap between UK policy and practice (Skerritt, 2023).

Across these different contexts, these developments represent significant opportunities to create new structures, relationships and communication channels to bridge the gap between research and practice in education. However, beyond funding to initiate and sustain these partnerships, funding should also be available to robustly evaluate these partnerships, to share experiences and best practice and to understand whether there is added value from this way of working (e.g., Bryk et al., 2023). This added value may be to educational research, practice and/or policy (depending on the original remit). Evaluations of this type would also allow those working in this area to gain insights into unsuccessful or less successful research‐practice partnerships and understand the reasons why these have failed to achieve the outcomes or outputs originally intended (Cooper et al., 2020).

### Training

The principles and practices involved in effectively co‐producing research typically require individuals to navigate new and uncertain social dynamics and ways of working (Coburn & Penuel, 2016; Skipper & Pepler, 2021) which their degrees and training may not have prepared them for (Denner et al., 2019). For example, in the absence of a broker or other individual independent from research and practice, university researchers often adopt the role of project facilitators, responsible for coordinating and integrating input from both research and practice (McGeown et al., 2023), while practice‐based professionals may have to quickly familiarize themselves with research processes and new terminology. To upskill and support the professional development of both, training is likely to include support for effective communication, values and principles, required research/practice knowledge and terminology, ethical and legal considerations, the promotion of innovation and learning from ‘best practice’ (McGeown, 2023). Beyond training however, actual experience working in research‐practice partnerships, and opportunities to reflect on these, will also be essential to develop individuals' skills and expertise. Further, beyond training for researchers and practitioners, educating brokers, that is, individuals to facilitate communication and collaboration between researchers and practitioners, could also be key for navigating the research‐practice‐policy nexus (Booker et al., 2019; Meyer et al., 2023; Skerritt, 2023) particularly if navigating more challenging, but necessary, collaborative projects.

Across numerous European contexts, and in different ways, there are growing efforts to create learning opportunities to better equip individuals with the necessary skills, expertise and experiences for engaging effectively in research‐practice partnerships. For example, in Sweden, at MDU University, a course is being integrated into all undergraduate programs, focusing on collaborative efforts between various stakeholders, such as industry, higher education institutions, and welfare organizations (Sjölund, 2023). While in the United Kingdom, an increasing proportion of PhD funding schemes aim to support the next generation of researchers with the skills and experiences to work successfully in research‐practice partnerships, with non‐academic partners often providing placements, project input and/or supervisory support (UKRI, 2024).

Beyond support for both undergraduate and postgraduate students, there is an increasing focus on educating brokers to act as intermediaries between research and practice. For example, the German Leibniz Institute for Research and Information in Education (DIPF) recently launched the Peers4Practice project. Funded by the Robert Bosch Foundation, to cultivate brokering skills among education researchers and school practitioners (Schlicht‐Schmälzle et al., 2024) this programme pairs early career researchers with early career teachers to develop sustainable skills for effective research‐practice collaboration over an 18‐month period. During this time, participants engage in joint classroom planning, reflections, and collaborative projects, aiming to enhance interdisciplinary communication and cooperation, translate and contextualize research findings for practical application and navigate the distinct epistemologies of research and practice (Schlicht‐Schmälzle et al., 2024).

In addition, individuals involved in research‐practice partnerships need to have the knowledge, experience and expertise to be able to contribute meaningfully and productively to research‐practice partnerships, but also need to have the dispositions to enter into these in the spirit of ‘mutuality’ so that all members can share their knowledge and contribute to the process and outcomes (Bevins & Price, 2014; Penuel, 2023).

As university‐based researchers are increasingly adopting, or being introduced to, research‐practice partnerships, this seems an appropriate time to think carefully and comprehensively about the types of training which will result in more positive and productive partnerships. The examples above illustrate training based primarily within University (Sweden) or real‐world contexts, either through short‐term placements (UK) or more sustained collaborative working (Germany). The most effective training for researchers and practice professionals is likely to integrate more formal instruction/learning (to benefit from others' past experience) with personal experience working within RPPs themselves, ideally with researchers and practice professionals learning together throughout and thus better understanding each other's contexts, knowledge, experience and interests/priorities. However, the reality is that funding, time, and existing infrastructures are all likely to put pressures and restrictions on the types of training available and how and where it is delivered.

### Incentives

A key concern regarding the initiation and sustainability of research practice partnerships is a lack of incentives for those in both research and practice to engage within them (Desimone et al., 2016). For example, for researchers, the additional time taken to initiate, nurture and sustain research‐practice partnerships can conflict with other academic priorities, for example, gaining tenure, or publishing more traditional research, which has historically, and in many instances continues to hold more weight and professional recognition among academic peers (Arce‐Trigatti et al., 2018). In addition, the current incentive systems for collaborative research (e.g., external funding schemes requesting involvement of research users) may still be problematic if they prioritize high‐quality research outputs (i.e., peer reviewed articles) over stakeholder/practice‐partner needs (e.g., research‐informed teacher resources). To address this issue, incentive systems for researchers need to align more closely with stakeholder needs, ensuring project outputs are meaningful and usable.

This is reflected, to some extent, within the Coalition for Advancing Research Assessment (CoARA, 2022) an ambitious and visionary movement committed to reform in research assessment. With signatories from over 800 organizations (e.g., universities, funders, professional bodies) from across the world, CoARA is committed to ‘reforming the methods and processes by which research, researchers, and research organisations are evaluated’ (CoARA, 2022). Recognizing the weighting typically placed traditional research outputs (peer‐reviewed journal articles) and metrics of success (e.g., journal impact factors, h‐index), CoARA supports greater recognition of diverse research outputs and practices. In doing so, it advocates for qualitative judgement supported by responsible use of quantitative indicators, aligning with the need for more relevant and usable research outcomes. Within the context of research‐practice partnerships, CoARA can enhance incentives for collaborative research by promoting relevance, inclusivity and practical impact.

Further examples of incentives are also evident at national levels, for example, in the United Kingdom, as previously highlighted, research council funding calls have increasing stressed the importance of ‘stakeholder’ or ‘user’ engagement to both conceptualize and conduct research (e.g., ESRC), while the UK's Research Excellence Framework has increased its weighted allocation of funding to universities on the basis of ‘impact’ and in the next REF allocation of funding will be based on ‘engagement and impact’. In reflection of this, UK university selection, recruitment and promotion processes are increasingly valuing, and actively seeking, knowledge exchange, engagement and impact experience beyond academia as part of their processes.

However, beyond incentives for researchers, it is essential that incentives are also considered for practice professionals. Incentives may include support for professional learning and development (Steel et al., 2021), attracting additional income or resources to their school or organizations (Desimone et al., 2016), or having easier access to research to inform their practice (Desimone et al., 2016).

While incentives are likely to differ for researchers and practice professionals, this is unlikely to be problematic if they are incentivizing the same activities and outcomes within the same timeframe. However, challenges will exist if differing or misaligned priorities, needs and timelines are incentivized—and these cannot be reconciled. In addition, an unintended consequence of extrinsic/external incentives is that they may encourage tokenistic partnerships, for example, named external partners on grant applications who have been invited to make little intellectual contribution but are added to increase chances of funding success. Indeed, when creating incentives, theories of motivation which make the distinction between intrinsic/internally driven motivators (e.g., personal value, satisfaction) and extrinsic/externally driven motivators (e.g., external recognition, rewards) (Ryan & Deci, 2000) are relevant and may provide useful insights into the potential implications of different types of incentives.

To summarize, the last decade has seen considerable shifts in universities' research cultures, and this trajectory is set to continue. Enablers for positive systemic change include due consideration to *infrastructure* to initiate, facilitate and sustain positive, effective and inclusive partnerships, *funding* to allow individuals to engage sufficiently and meaningfully to achieve positive and mutually beneficial outcomes, *training* for knowledge and skill development, and *incentives* to motivate collaborative working. Indeed, if research‐practice partnerships are to become a part of university research ecosystems, policies, infrastructure and funding will need to continue to reform to support collaborative research; evaluation criteria will need to continue to be expanded and adopted to include practical relevance and stakeholder engagement; and training and incentives to motivate and upskill individuals are needed. In many ways, the gap between educational research and practice has never been narrower (MacPherson, 2023), yet there is still considerable progress to be made.

## LIMITATIONS

This research article is not a systematic review and is intended to draw upon both academic research and grey literature considered to be of most value and interest to researchers, policy‐makers and professionals interested in research‐practice partnerships and to offer both research and practical insights. For those interested in a systematic review of research‐practice partnerships, please see Sjölund et al. (2022). For the purposes of this article, the authors selected examples across a range of European contexts to exemplify each of the enablers, to illustrate diversity in terms of how these enablers can be enacted. However, these practical insights are limited to those countries in which the authors are working, or collaborating with others, and a broader understanding across other countries would add considerable value. Further, countries vary considerably in terms of their educational policy landscape and research‐policy interactions, and this is likely to influence whether and how research‐practice partnerships are introduced and embedded at a national level. High policy commitment to educational research and high levels of engagement between educational policy and research are likely to be supportive of the different enablers for research‐practice partnerships discussed here. Further, whether education policy creates centralized or decentralized structures is also likely to significantly influence research‐practice partnerships infrastructure. The position of policy is therefore essential to consider within the context of research‐practice partnerships, and needs to be considered in future work (see Skerritt, 2023). Finally, the four enablers discussed in this article were identified by drawing upon research literature, grey literature, and practical knowledge and experience, and were considered to best reflect the enablers required to initiate, develop, sustain and evaluate high‐quality partnerships. However, as our knowledge and understanding of research‐practice partnerships continue to evolve, it will be important to revisit these enablers to ensure we are orientating our thinking, planning and resources optimally, to ensure research‐practice partnerships are able to achieve their potential within our research ecosystems.

## CONCLUSION

Research‐practice partnerships are dependent upon the engagement and coordination of multiple and diverse professionals, spanning different organizations and sectors to work towards an agreed shared goal, for example, educational improvement or reducing educational inequalities. This is complex work and not without challenges, yet in different international contexts, the growing use of research‐practice partnerships to inform university‐based research agendas and practices is becoming increasingly widespread and embedded—and this trajectory is set to continue. Research‐practice partnerships encourage us to reflect on the purposes of university research—who is research for, what does research excellence look like and who judges the quality of research? This article aimed to prompt thinking, discussion and action to promote and successfully navigate changes in our university research ecosystems. Research‐practice partnerships have implications for the research cultures and environments of our universities, for our outputs, and most importantly, for our potential to make a more meaningful and positive contribution to the lives and learning of children and young people.

## AUTHOR CONTRIBUTIONS

**Sarah McGeown:** Conceptualization; writing – original draft. **Simon Sjölund:** Writing – original draft.

## CONFLICT OF INTEREST STATEMENT

The authors report no conflicts of interest.

## Data Availability

There is no data associated with this manuscript.
